# Overexpression of the E2F target gene *CENPI* promotes chromosome instability and predicts poor prognosis in estrogen receptor-positive breast cancer

**DOI:** 10.18632/oncotarget.19131

**Published:** 2017-07-10

**Authors:** Pulari U. Thangavelu, Cheng-Yu Lin, Srividya Vaidyanathan, Thu H.M. Nguyen, Eloise Dray, Pascal H.G. Duijf

**Affiliations:** ^1^ University of Queensland Diamantina Institute, The University of Queensland, Translational Research Institute, Brisbane, QLD, Australia; ^2^ Institute of Health and Biomedical Innovation, Queensland University of Technology, Translational Research Institute, Brisbane, QLD, Australia

**Keywords:** breast cancer, prognosis, chromosome instability, aneuploidy, CENPI

## Abstract

During cell division, chromosome segregation is facilitated by the mitotic checkpoint, or spindle assembly checkpoint (SAC), which ensures correct kinetochore-microtubule attachments and prevents premature sister-chromatid separation. It is well established that misexpression of SAC components on the outer kinetochores promotes chromosome instability (CIN) and tumorigenesis. Here, we study the expression of CENP-I, a key component of the HIKM complex at the inner kinetochores, in breast cancer, including ductal, lobular, medullary and male breast carcinomas. CENPI mRNA and protein levels are significantly elevated in estrogen receptor-positive (ER+) but not in estrogen receptor-negative (ER-) breast carcinoma. Well-established prognostic tests indicate that CENPI overexpression constitutes a powerful independent marker for poor patient prognosis and survival in ER+ breast cancer. We further demonstrate that *CENPI* is an E2F target gene. Consistently, it is overexpressed in *RB1*-deficient breast cancers. However, CENP-I overexpression is not purely due to cell cycle-associated expression. In ER+ breast cancer cells, CENP-I overexpression promotes CIN, especially chromosome gains. In addition, in ER+ breast carcinomas the degree of CENPI overexpression is proportional to the level of aneuploidy and CENPI overexpression is one of the strongest markers for CIN identified to date. Our results indicate that overexpression of the inner kinetochore protein CENP-I promotes CIN and forecasts poor prognosis for ER+ breast cancer patients. These observations provide novel mechanistic insights and have important implications for breast cancer diagnostics and potentially therapeutic targeting.

## INTRODUCTION

Checkpoints are essential in cell cycle regulation. The mitotic checkpoint, or spindle assembly checkpoint (SAC), ensures that chromosomes segregate accurately during mitosis. While defects in the SAC lead to chromosome missegregation, mitotic slippage or apoptosis *in vitro* and *in vivo* and promote tumorigenesis [1, 2], the SAC is rarely defective in cancer cells [1–4]. It is more frequently overactivated, a phenomenon that also promotes chromosome instability (CIN) and cancer progression *in vivo* [1, 5]. Defects in major tumor suppressor pathways cause ‘oncogene-induced mitotic stress’, leading to SAC hyperactivation and CIN [2, 6, 7].

At the centromeres, kinetochores establish a platform for microtubule attachments, which are critical for faithful chromosome segregation. These interactions occur at the outer kinetochore, which is also the primary site of SAC signaling. Not surprisingly, therefore, a large body of research has focused on understanding the molecular mechanisms of SAC function, in particular studying key signaling components and effectors such as MAD1, MAD2, BUB1, BUBR1, TTK, CDC20 and APC/C. While these SAC proteins are important in controlling chromosome segregation [8], recent studies imply that various other inner and outer kinetochore components are required for the correct establishment of microtubule-kinetochore interactions and SAC function, thereby indirectly controlling chromosome segregation. For instance, the formation of a tetrameric structure consisting of the centromere proteins CENP-H, CENP-I, CENP-K and CENP-M, also termed HIKM complex, is crucial for the formation of efficient and correct microtubule attachments [9]. Although individual HIKM components may not be directly involved in microtubule-kinetochore interactions, they are essential for their efficient establishment and SAC function [10].

CENP-I is of particular interest because it links the inner and outer kinetochore via the formation of a tri-laminar structure regulated by the histone H3 variant CENP-A. Mutations in the N-terminal tail of CENP-A reduce the localization of CENP-I to the outer kinetochore [11]. In turn, mutations affecting CENP-I and CENP-M interactions disrupt functionality of the HIKM complex [9]. Establishment of a strong and mature kinetochore-microtubule connection is ensured by the RZZ complex (Rod, Zwilch and ZW10 proteins), which interacts with MAD1. CENP-I stabilizes RZZ-MAD1 binding to the kinetochore by inhibiting their removal through dynein stripping [12]. These interactions are crucial for the correct segregation of chromosomes [12].

Loss-of-function experiments have demonstrated that CENP-I is required for timely progression through G_2_ phase and mitosis and for the BUB1-dependent localization of MAD1, MAD2 and CENP-F to the kinetochore [13, 14]. CENP-I depletion leads to aberrant centromere assembly and integrity, the formation of monotelic microtubule-kinetochore attachments, a defective SAC and CIN [10, 13, 15–17].

CIN promotes cancer progression [1, 2] and more than two-thirds of all solid cancers are aneuploid [18]. Here, we aimed to investigate CENP-I expression in breast cancer. We find that CENP-I is overexpressed in estrogen receptor-positive (ER+) but not in estrogen receptor-negative (ER-) breast carcinomas. For the former, it constitutes a strong independent prognostic marker. In addition, *CENPI* is an E2F target gene, whose overexpression causes CIN *in vitro*. Finally, we find that CENPI overexpression is a more powerful marker for CIN in ER+ breast cancers than most well established CIN markers.

## RESULTS

### CENP-I is overexpressed in breast carcinoma

**Figure 1:**
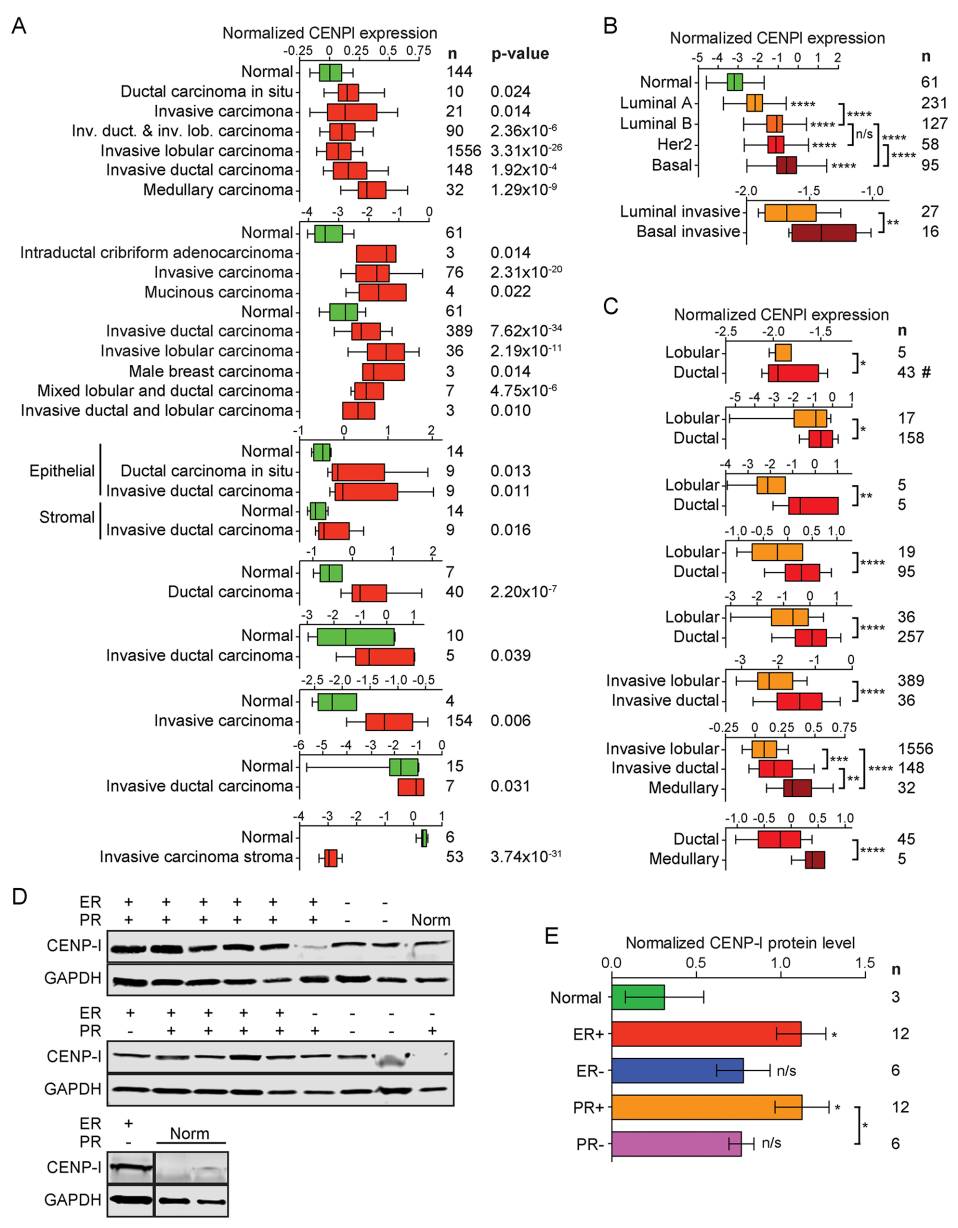
CENP-I mRNA and protein levels are elevated in ER+ breast cancer **(A)** Normalized CENPI mRNA expression in breast cancer compared to normal breast tissue. Data are derived from studies: [19–21, 43–47]. **(B)** Normalized CENPI mRNA expression in breast cancer molecular subtypes. Data are derived from studies: [21, 22]. **(C)** Normalized CENPI mRNA expression in breast cancer histological subtypes. Data are derived from studies: [21, 43, 45, 48–51]. **(D)** Western blots of primary normal (Norm) and breast carcinoma tissue samples with estrogen receptor (ER) and progesterone receptor (PR) status as indicated. **(E)** Quantification of CENP-I protein levels normalized to GAPDH protein levels in primary breast tumor and control breast tissue samples using the Western blots shown in (D). All p values: t-test.

We compared CENPI mRNA expression levels in 2664 breast cancer samples to those in 269 normal control breast tissue samples using 22 previously published datasets (see Methods). In 21 of these, CENPI was significantly overexpressed in tumor samples compared to normal breast tissue (Figure 1A). These breast cancer samples included ductal, lobular and medullary carcinomas, as well as male breast carcinomas, and they ranged from localized *in situ* lesions to invasive tumors. The only dataset that showed significant CENPI underexpression compared tumor stroma – rather than tumor *per se* – to normal tissue [19] (Figure 1A). In contrast, another study found that CENPI levels are increased in tumor stroma [20] (Figure 1A). Thus, while it is unclear whether CENPI levels are typically abnormal in breast cancer stromal cells, 20 out of 20 studies indicate that breast carcinoma intrinsic CENPI mRNA levels are significantly increased (Figure 1A).

We next used the TCGA breast cancer RNAseq dataset [21] to evaluate CENPI mRNA expression in the four well-established molecular subtypes: luminal A, luminal B, Her2-type and basal-like breast cancers. This revealed that the degree of CENPI mRNA overexpression correlated with clinical outcome, as luminal A tumors, which have the most favorable prognosis, show the lowest degree of overexpression, while the most malignant, basal-like breast cancers show the highest level of CENPI overexpression (Figure 1B, top). These results are consistent with significantly elevated CENPI levels in basal tumors as compared to luminal cancers in another dataset [22] (Figure 1B, bottom).

Data from seven independent studies show that CENPI mRNA levels are significantly higher in ductal breast carcinomas than in lobular breast carcinomas (Figure 1C). In turn, medullary breast carcinomas show significantly elevated CENPI mRNA levels compared to ductal carcinomas (Figure 1C).

To further investigate the above observations, we studied CENP-I protein levels in primary breast tumor and normal breast tissue samples using Western blot analysis. This indicated that, compared to normal control tissue, CENP-I levels are significantly higher in ER+ and progesterone receptor-positive (PR+) tumors (p=0.0225 and p=0.0212, respectively, unpaired t-test; Figure 1D, 1E). However, CENP-I levels are not significantly elevated in ER- or progesterone receptor-negative (PR-) tumors (p=0.0864, p=0.0901). The development of different breast cancer subtypes is strongly influenced by hormones and the status of ER and PR expression crucially dictates prognosis and guides treatment approaches. Our observation that CENP-I is overexpressed in hormone receptor-positive but not in hormone receptor-negative breast cancers is therefore of considerable clinical importance.

### CENPI overexpression is an independent marker for poor prognosis in ER+ breast cancer

Data from 24 independent studies were pooled (see Methods) to evaluate whether CENPI mRNA overexpression could serve as a biomarker for poor patient prognosis. Using univariate Cox proportional hazard analysis on 3826 breast cancer samples, we demonstrated that CENPI overexpression is a strong marker for poor patient prognosis (p=6.13×10^-10^) (Table 1).

**Table 1:** CENP-I overexpression is a strong independent prognostic marker for ER+ breast cancer

Type	No. of patients	HR (95% CI)	Prognostic strength		Adjuvant!^a^		Nottingham index^a^	
			p Value	p Value summary	p Value	p value summary	p Value	p Value summary
All	3826	1.26 (1.17-1.35)	6.13×10^-10^	****	0.0239	*	0.0421	*
ER+	2757	1.34 (1.22-1.47)	1.74×10^-9^	****	0.0313	*	0.0375	*
ER-	1039	1.02 (0.90-1.16)	0.7724	n/s	0.6375	n/s	0.6224	n/s

^a^ Calculated as described in [21] and [22], respectively.

We also assessed the prognostic strength of CENPI mRNA expression in ER+ and ER- breast cancers separately. Consistent with our previous findings, the prognostic power of CENPI overexpression was highly significant for ER+ but not for ER- breast cancers (Table 1).

Various clinical tests have been developed to predict breast cancer patient prognosis. Adjuvant! Online and the Nottingham Prognostic Index are among the most well-established ones. Adjuvant! Online is a computer program that takes various clinical parameters into account in its projection of breast cancer patient outcome with the goal to assist in decision making related to the use of adjuvant therapies [23]. The Nottingham Prognostic Index helps predict post-surgery outcome by including tumor size, histologic grade and the number of positive lymph nodes [24]. Using the same combined datasets, CENPI overexpression was subjected to these tests in order to more stringently determine its potential as a biomarker, as described [23, 24]. This continued to provide significant prognostic strength for all breast cancers combined and for ER+ cancers but not for ER- breast cancers (Table 1). Thus, these two tests independently indicate that CENPI overexpression is a strong independent marker for ER+ breast cancer patient prognosis.

### CENPI overexpression is a marker for poor patient survival in ER+ breast cancer

**Figure 2:**
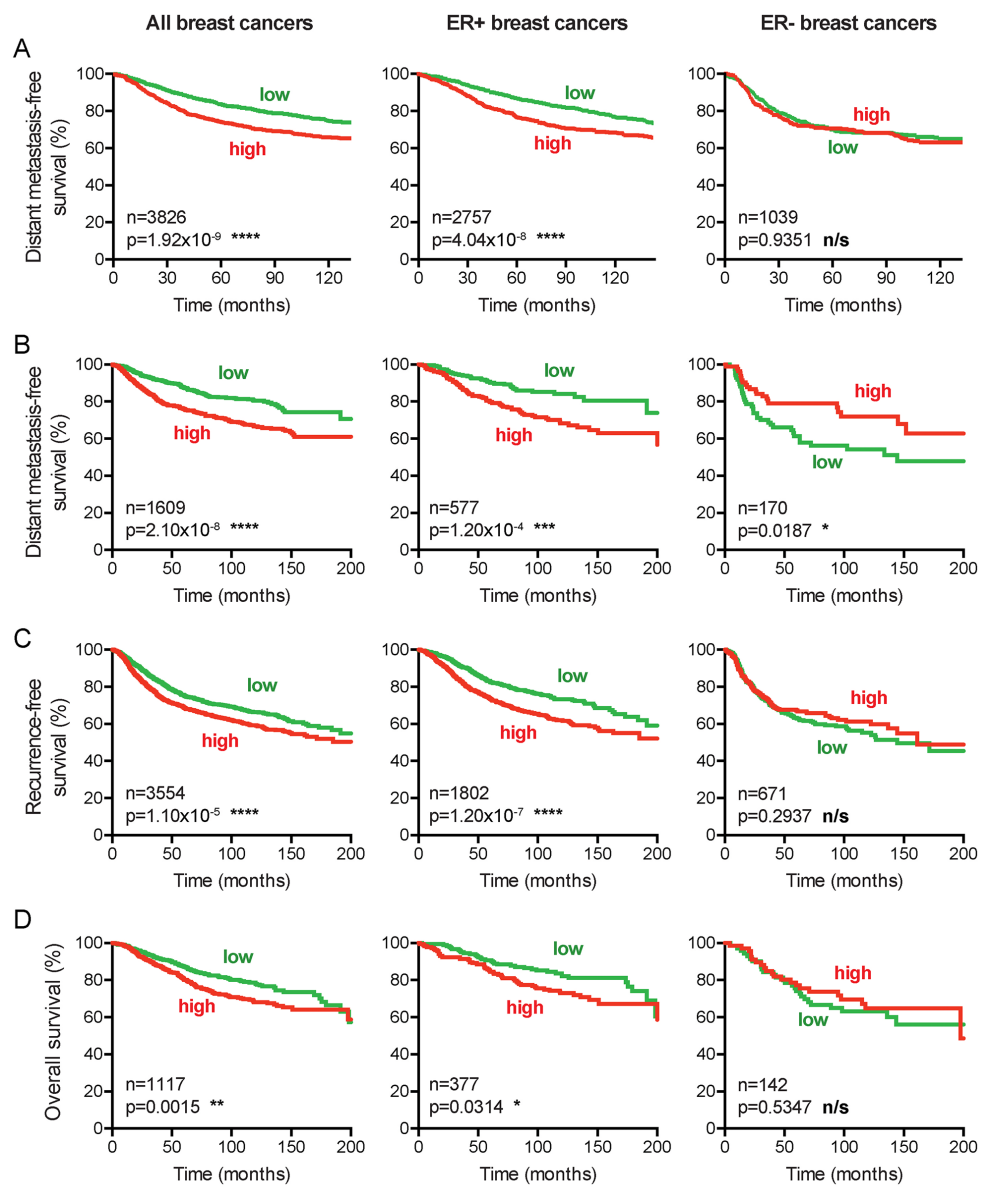
CENPI overexpression is a marker for poor survival in ER+ but not ER- breast cancer **(A)** Distant metastasis-free survival curves of patients from 24 combined datasets (see Methods). Patients were split into high and low CENPI mRNA expression groups using the median CENPI expression level as the cut-off. P-values: log-rank test. **(B-D)** Distant metastasis-free, recurrence-free and overall survival curves, respectively, of patients with high and low expression levels of CENPI, determined as previously described [25]. P-values: log-rank test.

To further corroborate the prognostic value of CENPI expression, we evaluated distant metastasis-free survival of the breast cancer patients using the pooled datasets. Consistent with our previous findings, for all breast cancers combined and for ER+ cancers separately, high CENPI mRNA levels provide a significantly worse prognosis than low CENPI mRNA levels, while no significant difference was observed for ER- breast cancers (Figure 2A). To further validate these findings, we also performed survival analysis as previously described [25]. This enabled us to not only assess distant metastasis-free survival, but also recurrence-free survival and overall survival. These analyses confirmed the above findings (Figure 2B–2D). Interestingly, however, in this dataset lower CENPI levels conferred poorer distant metastasis-free survival for ER- breast cancers (p=0.0187, log-rank test; Figure 2B). Taken together, we conclude that elevated CENPI levels provide a significantly poor patient prognosis for ER+ breast cancers but not for ER- cancers.

### Mechanism of CENP-I overexpression

**Figure 3:**
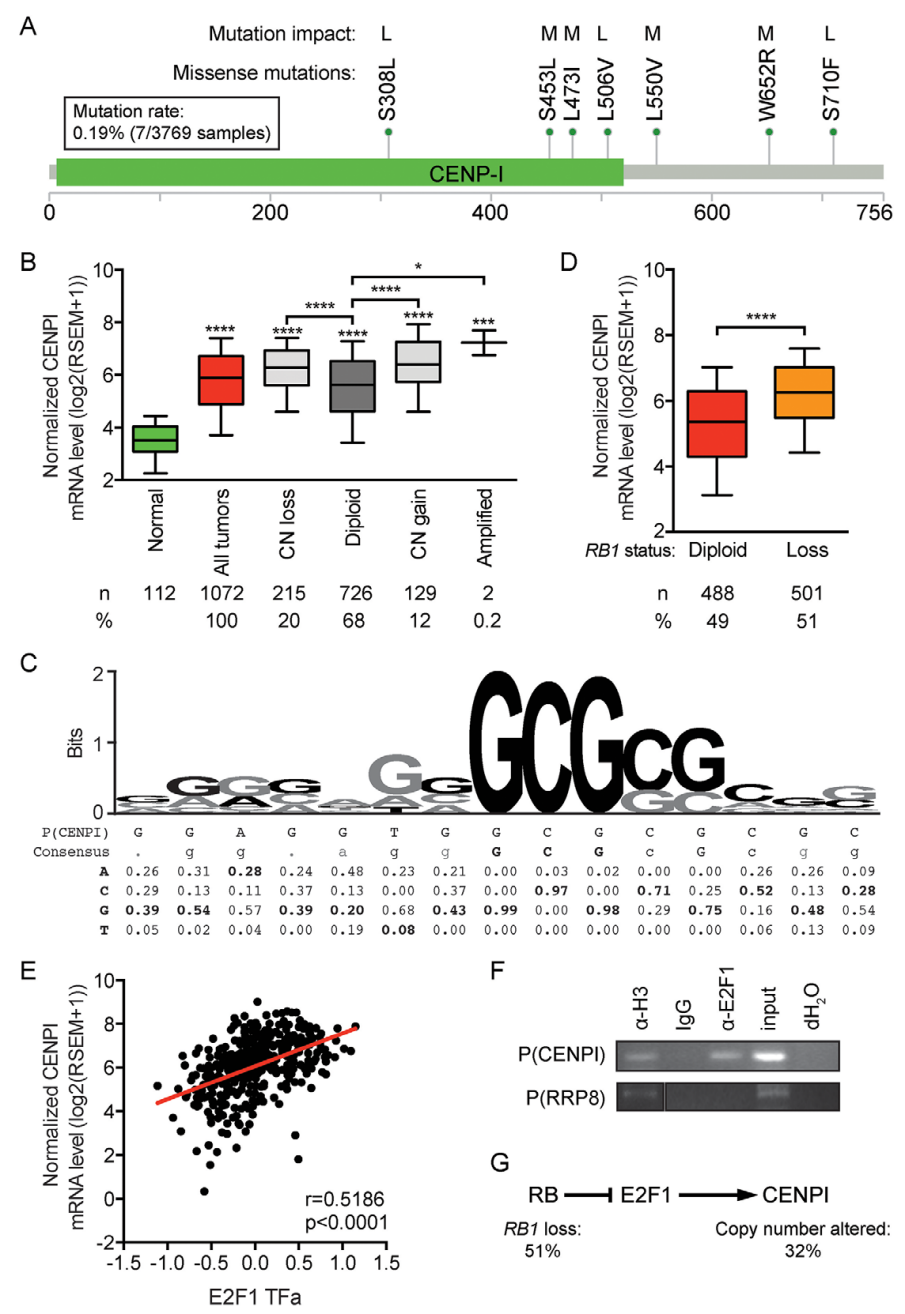
*CENPI* is an E2F target gene, indicating that CENPI overexpression in breast cancer is primarily caused by RB pathway defects **(A)** Mutations identified in 3769 breast cancer samples from 5 datasets (see Methods), as described [56, 57]. Each mutation was identified only once. The functional impact of the mutations was assessed as described [26] with all identified mutations predicted to have low (L) or medium (M) impact on protein function. The image was obtained by and modified from [56] and [57]. Scale bar indicates amino acid numbers. **(B)** CENPI mRNA expression levels in normal control breast tissue and breast carcinomas for *CENPI* allelic copy number categories, as indicated. Data are derived from the TCGA RNAseq and SNP6 microarray datasets [21]. **(C)** Sequence logo of the E2F1 DNA binding site with consensus sequence and nucleotide frequencies at each position below [27]. The putative E2F1 DNA binding site in the CENPI promoter, P(CENPI), located from positions -127 to -113 upstream of the CENPI transcription start site, was aligned below and overlaid in black font on the sequence logo above. **(D)** Normalized CENPI mRNA levels in breast carcinoma samples diploid and with copy number loss of the *RB1* allele. P-value: Mann Whitney *U* test. **(E)** CENPI mRNA levels compared to inferred E2F1 transcription factor activity, computed as described [28, 29]. P-value: Spearman correlation. **(F)** Chromatin immunoprecipitation (ChIP) using IgG, histone H3-specific (α-H3) and E2F1-specific (α-E2F1) antibodies. PCRs were performed on the CENPI promoter, P(CENPI), and the RRP8 promoter, P(RRP8). IgG and dH_2_O served as negative controls, α-H3 and input served as positive controls and P(RRP8) served as negative control for α-E2F1. **(G)** Retinoblastoma (RB) pathway showing *CENPI* as an E2F1 target gene. *RB1* loss and, to a lesser extent, *CENPI* allelic copy number alterations contribute to CENPI overexpression in breast cancer.

To assess how CENP-I may be overexpressed in breast cancer, we first considered the possibility that mutations could stabilize CENP-I mRNA or protein. In five large datasets (see Methods), together comprising 3769 samples, only 7 mutations – all missense mutations – were identified. This equates to a mutation rate of 0.19% (Figure 3A). In addition, an algorithm to assess these mutations’ impact on protein function, only predicted a low to medium effect of these few mutations [26] (Figure 3A). These data indicate that CENPI mutations do not contribute to its widespread overexpression.

Next, we assessed whether CENPI allelic copy number gains or amplifications contributed to CENP-I overexpression using the TCGA breast cancer RNAseq and SNP6 array data [21]. Twelve percent of the tumors showed CENPI copy number gains or amplifications and the CENPI mRNA levels in these tumors – as well as in tumors with copy number loss – showed significantly elevated mRNA levels compared to CENPI diploid tumors (p<0.0001, p<0.0001, p=0.0257, respectively, Mann Whitney *U* test) (Figure 3B). However, compared to normal tissue, CENPI mRNA levels were significantly higher even in CENPI diploid cancers (p<0.0001) (Figure 3B). Thus, while CENPI mRNA levels are elevated in breast carcinomas with CENPI copy number changes, this does not fully account for the observed CENPI overexpression.

The above data suggest that another mechanism contributes more profoundly to CENPI overexpression in breast cancer. Interestingly, examination of the CENPI promoter sequence identified a potential E2F1 binding site from positions -127 to -113 relative to the CENPI transcription start site (Figure 3C). Alignment of this sequence with the E2F1 DNA binding consensus [27], indicated that 5 out of 5 of the most important core nucleotides occurred at the highest consensus frequencies. The same was true for three flanking nucleotides and of the remaining seven, all of which were much less critical, another three matched the most frequent consensus nucleotides. This suggested that *CENPI* could be an E2F1 target gene. In normal cells, the Retinoblastoma (Rb) protein restrains E2F1 transcription factor activity via direct protein-protein interaction. Hence, *RB1* loss, a common event in breast cancer, results in increased mRNA levels of E2F1 target genes. Consistent with this notion, CENPI mRNA levels are significantly elevated in breast cancers with *RB1* loss (p<0.0001, Mann-Whitney *U* test; Figure 3D). Moreover, in TCGA breast tumors, the predicted E2F1 transcription factor activity, inferred from the tumor sample’s protein expression profile using a trained affinity regression model [28, 29], positively correlates with CENPI mRNA level in the tumors (r=0.5186, p<0.0001, Spearman correlation; Figure 3E). Finally, chromatin immunoprecipitation (ChIP) assays using an E2F1-specific antibody demonstrated that E2F1 binds to the CENPI promoter but not to the RRP8 negative control promoter (Figure 3F). Thus, these data indicate that CENPI is an E2F1 target gene and strongly suggest that CENPI overexpression is widespread in breast cancer primarily due to frequent Rb pathway defects, while CENPI allelic copy number aberrations contribute less substantially (Figure 3G).

### CENPI is not overexpressed in breast cancer due to a proliferation-associated effect

**Figure 4:**
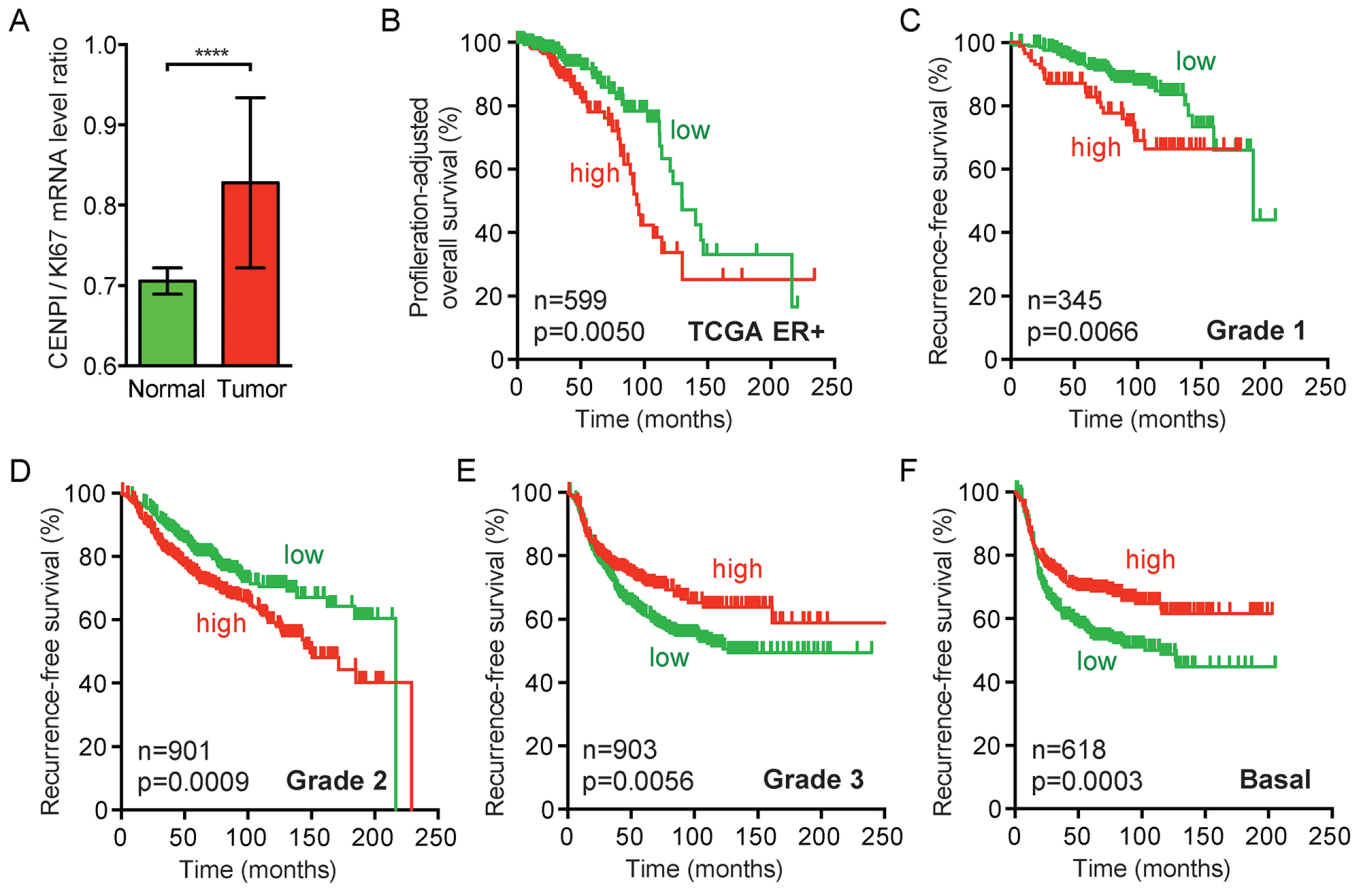
CENPI overexpression in ER+ breast cancer cannot be fully explained by a proliferation-associated effect **(A)** Bar graph of proliferation-adjusted CENPI mRNA levels in TCGA normal control and breast carcinoma samples. The ratio of CENPI mRNA level divided by MKI67 (KI67) mRNA level (CENPI/KI67) is plotted on the y-axis. P-value: Mann-Whitney *U* test. **(B)** Proliferation-adjusted survival curves of TCGA ER+ breast cancer patients. CENPI/KI67 ratios as in (A) were calculated for each sample and patients were split in high and low CENPI/KI67 ratio using the median ratio as a cut-off. P-value: log-rank test. **(C-F)** Recurrence-free survival curves as in Figure 2C for grade 1, grade 2, grade 3 and basal breast cancer patients, respectively. P-value: log-rank test.

Our identification of *CENPI* as an E2F1 target gene suggests that its expression is higher in cycling cells than in non-proliferating cells in G_0_/G_1_ stage of the cell cycle. This could mean that CENP-I is overexpressed in breast tumors merely due to the increased proliferation in tumor tissue compared to normal tissue. However, for a number of reasons, we believe that this is not the case. First, we observed that breast cancer cell lines express different protein levels of CENP-I, but these differences do not markedly change when their levels are compensated for by the protein levels of the proliferation marker PCNA (data not shown). Second, when we adjust the prognosis predictive power of CENPI expression, as calculated in Table 1, to a well-established proliferation gene expression signature [30], it remains strongly associated with poor prognosis for ER+ breast cancer but not for ER- breast cancer (p=0.0183 for all breast cancers, p=0.0067 for ER+ and p=0.5335 for ER- tumors). Third, CENPI mRNA levels are increased in ER+ breast cancers, even when compensated for by the proliferation marker KI67 (p<0.0001, Mann Whitney *U* test) (Figure 4A). Fourth, when we adjust the CENPI expression levels in ER+ breast cancer patients for cell proliferation by dividing these levels by the respective KI67 levels, patients with CENPI/KI67 levels above the median have a significantly poorer prognosis than those with CENPI/KI67 levels below the median (p=0.0050, log-rank test) (Figure 4B). Fifth, while ER- breast cancers are typically more aggressive and hence proliferate more rapidly compared to ER+ tumors and normal tissue, CENP-I protein levels are not significantly increased in these ER- cancers compared to normal control tissue (Figure 1D). Sixth, recurrence-free survival curves indicate that high CENPI expression forecasts poor prognosis for Grade 1 and Grade 2 breast cancer patients (p=0.0066 and p=0.0009, log-rank test), but the converse applies to Grade 3 and basal breast cancer patients, whose cancers are more aggressive and hence have the highest proliferation rates (p=0.0056 and p=0.0003) (Figure 4C–4F). We note, though, that the latter is probably partly explained by the fact that Grade 3 and basal breast cancers are typically ER- (see also Figure 2). Taken together, these observations indicate that CENPI overexpression in breast cancer cannot be explained by a proliferation-associated effect.

### CENP-I overexpression promotes chromosome instability and chromosome gains in ER+ breast cancer cells

**Figure 5:**
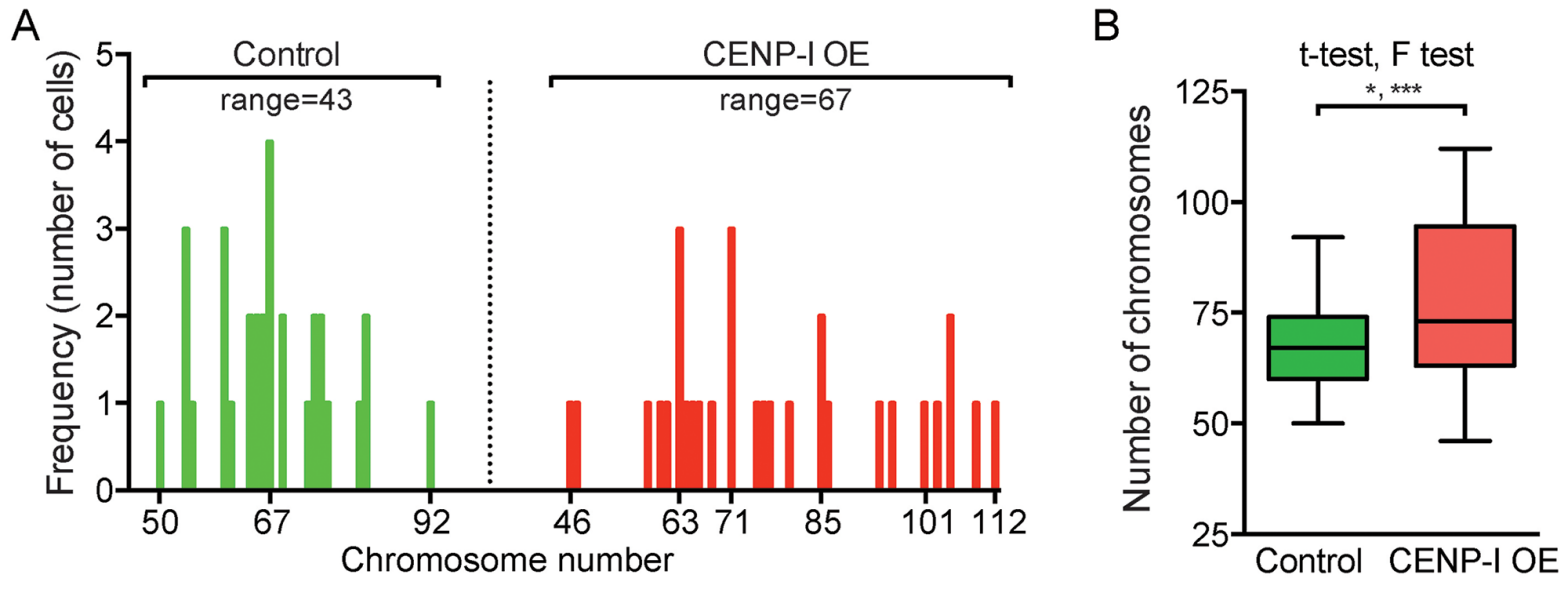
CENP-I overexpression promotes chromosome instability, in particular chromosome gains, in ER+ breast cancer cells **(A)** Histograms of ER+ MCF7 cells with indicated chromosome numbers. CENP-I overexpressing (OE) cells (right, n=30) are compared to non-CENP-I overexpressing control cells (left, n=31). **(B)** Bar graph of the chromosome numbers in MCF7 cells shown in (A). F test assesses whether the ranges of chromosome numbers differ; t-test assesses whether the means differ; *, p<0.05; ***, p<0.001.

We and others previously identified E2F target genes whose overexpression promotes chromosome instability (CIN) [5, 6, 31, 32]. To investigate whether CENP-I overexpression directly promotes chromosome missegregation, we overexpressed CENP-I in the ER+ breast cancer cell line MCF7 and compared the chromosome numbers in these cells to control MCF7 cells that did not overexpress CENP-I. Compared to control cells, CENP-I-overexpressing cells showed a broader range of chromosome numbers (50 to 92 [range=43] versus 46 to 112 [range=67]) (Figure 5A). This variance in chromosome numbers was statistically significantly different (p=0.0006, F test) (Figure 5B). This significantly broader range of chromosome numbers is a distinctive feature of CIN. We also found that the mean chromosome number in CENP-I-overexpressing cells was significantly higher than in control cells (p=0.0102, t-test) (Figure 5B). Thus, these results indicate that CENP-I overexpression promotes CIN, and in particular chromosome gains, in ER+ breast cancer cells.

### CENPI overexpression is a strong independent marker for chromosome instability in ER+ breast cancer

**Figure 6:**
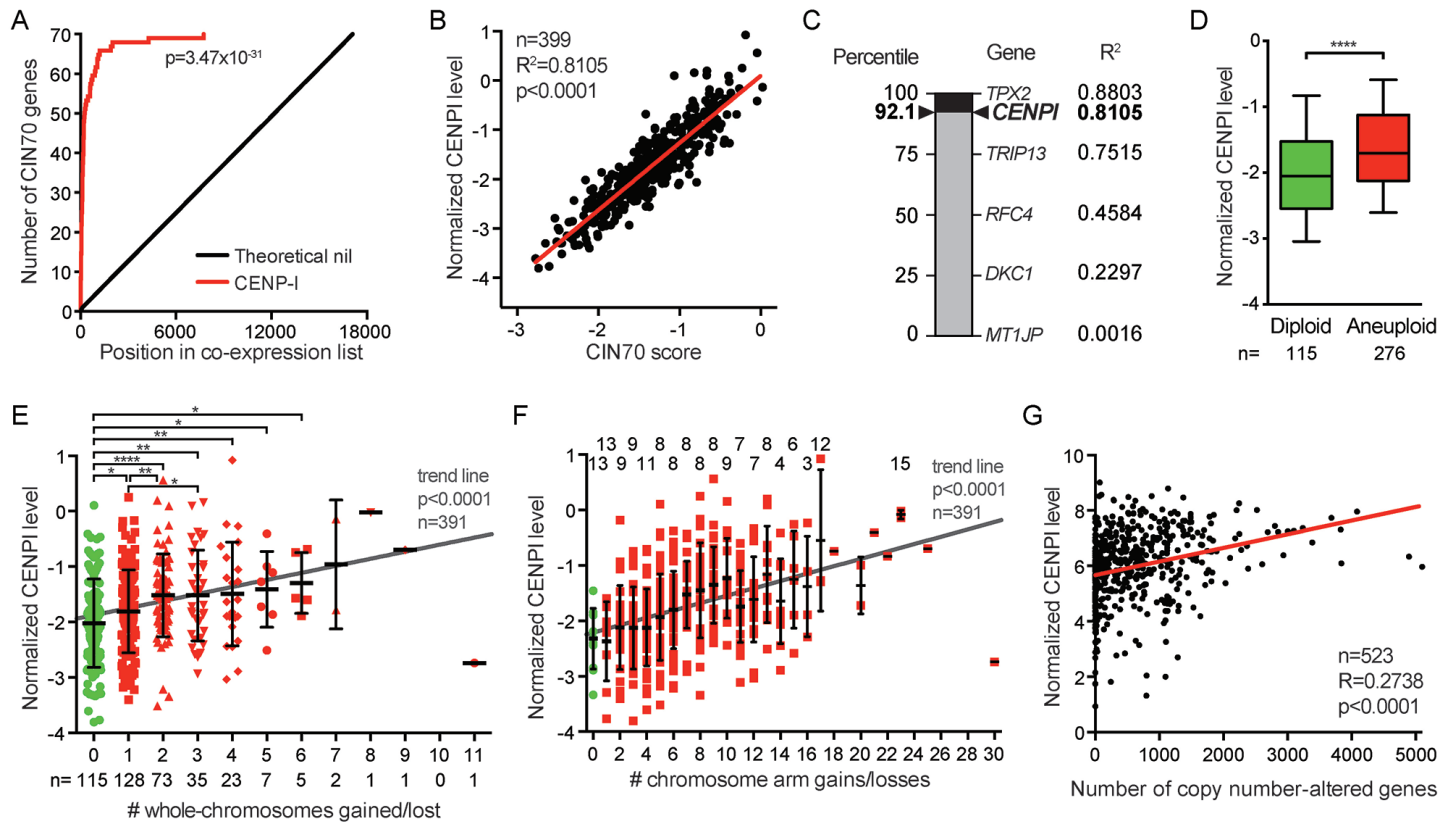
CENPI is a powerful marker for chromosome instability in ER+ breast cancer **(A)** Gene expression significance signature plot. The degree of co-expression of each gene with CENPI in TCGA ER+ breast cancers was determined by Pearson correlation and genes were ranked from highest to lowest correlation. The red line indicates the position of the number of CIN70 genes in the list, compared to a theoretical no-correlation line in black. P-value: log-rank test. **(B)** Scatter plot of CENPI mRNA expression level against the CIN70 score for chromosome instability in ER+ TCGA breast cancer samples. The regression line denotes the sum of least squares fit to the data points. P-value: Pearson correlation. **(C)** The strength of CENPI as a marker for chromosome instability is benchmarked to the performance of the 70 individual genes that contribute to the CIN70 signature, as determined by the R^2^. Percentiles are indicated on the left. Note that CENPI is not part of the CIN70 signature. **(D)** Box plot showing CENPI mRNA levels in diploid and aneuploid ER+ breast cancers. Whiskers show 10-90 percentiles. P-value <0.0001 (****), assessed by unpaired t-test. **(E)** Scatter dot plot comparing CENPI mRNA levels to the number of whole-chromosome gains and losses in ER+ breast tumors. Means with standard deviations are indicated, as well as significance levels as per unpaired t-tests: *, p<0.05; **, p<0.01; ****, p<0.0001. Trend line level of significance is assessed using the F-statistic. **(F)** Scatter dot plot, as in (E) for chromosome arm gains and losses. The numbers on top indicate with how many other groups in the graph each group shows a statistically significant difference at p<0.05, as per unpaired t-tests. **(G)** Scatter plot comparing CENPI mRNA level to the number of copy number-altered genes in each breast cancer sample. P-value: Pearson correlation.

We next used the TCGA breast cancer RNAseq dataset [21] to identify the genes that are most significantly co-expressed with CENPI in these cancers. Interestingly, this unbiased analysis showed that many of these genes are involved in chromosome segregation. In fact, all of the genes in the top 30 of this list have known roles in chromosome segregation and/or stability (Table 2). Interestingly, we noticed that 40% of these (12 of 30) are part of the CIN70 signature, a 70-gene expression signature that marks chromosome instability in human tumors [33] (Table 2). CENPI is not among these 70 genes. With 12 of the 70 CIN70 genes in the top 30 and 58 CIN70 genes among the remaining 17785 genes in the list, the CIN70 genes are highly significantly enriched in the top of the list (p<0.0001, Chi-square test). A more inclusive analysis, which compares the positions of the CIN70 genes in the CENPI co-expression list – ranked from highest to lowest Pearson’s correlation coefficient – to theoretical no-correlation ranks, confirmed a highly significant co-expression of CENPI and CIN70 genes (p=3.47×10^-31^, log-rank test; Figure 6A). This observation prompted us to explore the correlation between CENPI expression and CIN more directly by plotting the CIN70 scores of the TCGA ER+ breast cancer samples against their normalized CENPI levels. This revealed a very strong linear correlation between these two parameters (R^2^=0.8105, p<0.0001, Pearson correlation; Figure 6B).

**Table 2:** Genes most significantly co-expressed with CENP-I^a^

Rank	Gene^b^	Pearson’s coefficient
**1**	***TPX2***	**0.8549**
**2**	***KIF4A***	**0.8535**
3	*KIF4B*	0.8498
4	*EXO1*	0.8463
5	*BUB1*	0.8458
**6**	***MCM10***	**0.8445**
7	*DEPDC1B*	0.8303
8	*ERCC6L*	0.8293
9	*CENPA*	0.8179
**10**	***UBE2C***	**0.8177**
11	*FAM83D*	0.8169
12	*CCNA2*	0.8165
13	*DLG7*	0.8149
**14**	***MELK***	**0.8144**
15	*CDCA5*	0.8113
**16**	***TTK***	**0.8098**
17	*SPC25*	0.8094
**18**	***CDC2***	**0.8075**
19	*BUB1B*	0.8065
20	*CENPE*	0.8052
**21**	***CCNB2***	**0.8040**
**22**	***CCNB1***	**0.8009**
**23**	***NCAPH***	**0.8001**
24	*ARHGAP11A*	0.7998
25	*KIF23*	0.7986
26	*SHCBP1*	0.7973
**27**	***CEP55***	**0.7970**
**28**	***KIF20A***	**0.7961**
29	*NUSAP1*	0.7953
30	*RACGAP1*	0.7948

^a^ Calculated using TCGA breast cancer dataset [19].

^b^ CIN70 genes are highlighted in bold.

The tight correlation between CIN70 and CENPI expression led us to test the strength of CENPI overexpression as an independent marker for CIN in ER+ breast cancer. To do this, we computed how well the expression of the 70 individual members of the CIN70 signature correlated with the CIN70 score, as measured by their R^2^ values. With an R^2^ of 0.8803, TPX2 performed best, while some other CIN70 genes performed poorly (Figure 6C). With an R^2^ of 0.8105, CENPI ranks at the 92^th^ percentile in this range of well-established markers for chromosome instability (Figure 6B, 6C). This indicates that CENPI overexpression is a strong independent marker for chromosome instability in ER+ breast cancer.

Chromosome instability leads to aneuploidy, an abnormal chromosome number. We find that CENPI levels are significantly higher in aneuploid ER+ breast tumors than in diploid tumors (p<0.0001, t-test; Figure 6D). Further stratification of this indicates that among aneuploid tumors, CENPI levels also significantly increase with increased degrees of aneuploidy (p<0.05, t-tests; p<0.0001, F-test for trend line slope; Figure 6E).

Whole-chromosome instability (W-CIN) has been shown to promote structural chromosome instability (S-CIN), including gains and losses of fractions of chromosomes [34]. Consistently, CENPI levels increase along with an increase in the number of chromosome arm gains or losses in ER+ breast cancer (p<0.05, t-tests; p<0.0001, F-test for trend line slope; Figure 6F). In addition, there is a significant linear correlation between the level of CENPI mRNA expression and the number of copy number-altered genes (Pearson p<0.0001; Figure 6G). In contrast, there is no such correlation between the CENPI expression level and the number of non-synonymous mutations (Pearson p=0.7781; data not shown). Thus, CENPI overexpression causes CIN *in vitro* and strongly correlates with markers for both chromosome instability and aneuploidy in ER+ breast cancers.

## DISCUSSION

We find that CENPI overexpression is a marker for poor patient outcome in breast cancer. At the mRNA level, CENPI is overexpressed across all breast cancer subtypes. However, at the protein level CENP-I is overexpressed only in ER+ breast cancers. Consistently, CENPI overexpression negatively affects patient survival only for ER+ patients. In addition, CENPI overexpression is a marker for poor prognosis for ER+ but not ER- breast cancer, even when multiple key clinical parameters included in Adjuvant! Online and the Nottingham Prognostic Index are taken into account. We further show that CENPI overexpression in breast cancer is also proliferation-independent. Together, these data indicate that CENPI overexpression is a powerful independent marker for poor patient prognosis in ER+ breast cancer.

We identify the mechanism by which CENPI is overexpressed. The *CENPI* gene is located on the X chromosome [35]. This could suggest that CENPI overexpression is a consequence of aberrant X-inactivation. Some microscopic, genomic and epigenetic evidence supports this hypothesis [36–38]. In addition, in a mouse mammary tumor model, X-linked genes, including *Cenpi*, were found to be specifically overexpressed [39]. However, we also observed a significant increase in CENPI expression in male breast cancer (Figure 1A) [21] and we find that even CENPI allelic copy number increases only modestly increase CENPI mRNA levels (Figure 3B). This strongly suggests that another – gender-independent – mechanism of CENPI overexpression is far more important.

Indeed, we find that *CENPI* is a novel E2F1 target gene, thus placing its expression under direct control of the Retinoblastoma (Rb) pathway. Our findings that *RB1* loss and increased inferred E2F1 transcription factor activity are both associated with increased CENPI mRNA levels support this observation. Hormone receptor status strongly affects the prognostic strength of CENPI mRNA overexpression and CENP-I protein overexpression with their respectively being more powerful and higher in ER+ breast cancers. In fact, high CENPI expression consistently predicts poor prognosis in ER+ breast cancer patients, whereas high CENPI expression has either no prognostic power or forecasts better prognosis in ER- breast cancer patients (Figures 2 and 4). Interestingly, this phenomenon is characteristic for Rb pathway regulated genes, as it has been observed that ER+ breast cancers show a strong association between a high Rb-loss gene expression signature and poor patient prognosis, whereas the opposite is seen for ER- breast cancer patients [40]. Strikingly, this Rb-loss gene expression signature includes many genes that are also part of the CIN70 gene expression signature, as well as genes significantly co-expressed with CENPI (Table 2) [33, 40].

We also identify a key mechanism by which CENPI overexpression drives tumorigenesis. Similar to other E2F target genes, as we and others have previously shown [5, 6, 31, 32], we here find that CENPI overexpression promotes CIN, which facilitates tumor development and drug resistance in a variety of ways [1, 2]. Loss of CENPI expression had previously been shown to promote CIN *in vitro* [10, 13, 15–17]. We find here that in ER+ breast cancer cells, CENPI overexpression also promotes CIN (Figure 5). Importantly, the latter is more relevant in the context of breast cancer, as CENPI is frequently overexpressed, rather than underexpressed, in ER+ breast cancer. Together with our observation that CENPI overexpression is strongly associated with both aneuploidy and poor patient prognosis, this indicates that CENP-I overexpression promotes CIN during ER+ breast cancer development. This resembles the consequences of misexpression of a number of other mitotic regulators, whose reduced and increased expression both promote CIN, while only the latter is highly prevalent in cancers. For example, this has been observed for MAD2 and EMI1, each of which are both APC/C inhibitors and E2F targets [5, 32, 41, 42]. It has been proposed that overexpression of such mitotic regulators and the consequent genomic instability is caused by ‘oncogene-induced mitotic stress’ as a result of common defects in major tumor suppressor pathways [2, 6, 7]. Our data indicate that CENPI overexpression, as a result of Rb pathway defects, contributes to this as well.

CENPI is not part of the CIN70 gene expression signature for chromosome instability in human cancers [33]. Our study shows that CENPI ranks in the 92^nd^ percentile among these 70 well-established markers for CIN, indicating that CENPI overexpression is one of most powerful markers for CIN in ER+ breast cancer identified to date. Thus, inclusion of CENP-I in the CIN70 signature would increase its strength as a CIN marker, at least in ER+ breast cancer. More importantly, however, this observation is significant from a diagnostic and/or prognostic perspective, as it could aid in predicting clinical outcome of ER+ breast cancer patients.

## MATERIALS AND METHODS

### Gene expression analyses

Twenty-two published breast cancer datasets were used to compare CENPI mRNA expression between normal and tumor samples and between lobular, ductal and/or medullary breast cancer, as previously described [19–22, 43–52]. In these studies, normal control tissue may refer to healthy tissue from a cancer patient (matched control sample) or healthy tissue from a healthy control individual (unmatched control sample), as detailed in the cited literature. All datasets are available from the Gene Expression Omnibus (GEO): http://www.ncbi.nlm.nih.gov/geo and The Cancer Genome Atlas (TCGA): https://tcga-data.nci.nih.gov. For analysis of TCGA breast cancer samples, Agilent level 3 log2 lowess-normalized mRNA expression values were downloaded from the TCGA data portal to compare normalized CENPI mRNA expression levels of normal, tumor and/or breast cancer subtype, as indicated [21]. Unpaired t-tests were performed to assess whether differences were statistically significant. To determine which genes were most significantly co-expressed with CENPI, Pearson correlation coefficients of all genes were calculated using their normalized expression levels from the TCGA breast cancer dataset and genes were ranked from high to low according to their correlation coefficient with CENPI mRNA expression.

### Primary tissue processing

With approval from the Human Research Ethics Committee of the University of Queensland, frozen primary tissue samples, i.e., normal control breast tissues and breast tumor tissues, were obtained from the Wesley Research Institute Tissue Bank, Brisbane QLD, Australia. Tissues were processed as described [32] with minor modifications. Tissues were minced and 60-70mg was suspended in 200μl RIPA buffer without detergent (150mM NaCl, 50mM Tris-HCl, pH8.0) but with protease inhibitor cocktail (PIC; 1:500 v/v; Sigma P8340) and phosphatase inhibitors (0.1mM sodium orthovanadate, 10mM sodium fluoride, 25mM beta-glycerophosphate), and 0.40-0.45g Zirconia beads (Daintree, 1mm and 0.1mm in diameter) were added in a 1:1 ratio. Tissues were homogenized using a Precellys 24 high-throughput tissue homogenizer according to the manufacturer’s instructions. A detergent cocktail comprising of 1% Triton-X, 0.5% sodium deoxycholate and 0.1% SDS was added in a 1:4 ratio to the homogenized tissue and mixed by pipetting slowly. After a 10 min incubation on ice, the mixture was centrifuged for 30min at 13,200rpm at 4°C. The supernatant was used for Western blot analysis.

### Western blot analysis

A total of 20μg protein from primary breast tissue was subjected to SDS-PAGE. Proteins were resolved and separated in NuPAGE 3-8% Tris-Acetate protein gels (EA0378BOX, Thermo Fisher Scientific) at 100V for 2hrs and wet-transferred onto Immunobilin-FL PVDF membrane (0.45μm pore size, Merck Millipore) overnight at 30V and 4°C. Membranes were blocked with TBS-based Odyssey blocking buffer (Li-COR) for 2hr, washed 3×15min with TBS-T (TBS-0.1% Tween-20), incubated overnight at 4°C in blocking buffer with primary antibody, washed 3×5min with TBS-T, incubated with IRDye680- or IRDye800-labelled secondary antibody (Li-COR) for 1hr at room temperature (RT), washed 3×5min with TBS-T and 2×5min with TBS. Wet blots were scanned on an Odyssey CLX Imager (Li-COR). Primary antibodies used were: anti-CENPI antibody (Abcam, ab118796; 1:2300 dilution) and anti-GAPDH (G3PDH) (Trevigen, 2275-PC-100, 1:5000 dilution). Protein levels were quantified using Li-COR imaging software. Per sample, CENP-I level was normalized to that of GAPDH. Unpaired student t-tests were used to determine statistical significance between groups.

### Clinical prognostic tests

Univariate Cox proportional hazard analyses were used to calculate hazard ratios (HR) and 95% confidence intervals (CI) and determine whether CENPI mRNA overexpression was a significant prognostic marker for poor patient outcome, as defined by distant metastasis-free survival and as previously described [53, 54]. This calculated “prognostic strength” (HR with 95% CI) was also adjusted for clinical parameters included in Adjuvant! Online and the Nottingham prognostic index, as also previously described [23, 24] to assess the extent to which CENPI overexpression could independently serve as a prognostic marker.

### Survival analyses

For survival analysis on 24 independent datasets [53], patients were grouped into low and high expression, using the median expression as the cut-off, as described [53–55]. Additional survival analyses were performed using the Kaplan-Meier Plotter tool [25]. For the latter, survival curves were re-plotted in GraphPad Prism. For all comparisons of survival curves, log-rank Mantel-Cox tests were used to assess statistical significance.

### Mutation analysis

Mutations in *CENPI* were identified as described [56, 57]. The results from five studies were included in this analysis: Broad, Nature 2012; British Columbia, Nature 2012; Sanger, Nature 2012; TCGA, Provisional; METABRIC, Nature 2012 & Nat Commun 2016, as referenced [56, 57]. The total sample numbers with the number of mutated samples were 103 samples/0 *CENPI* mutations, 65/1, 100/1, 1105/5, 2399/0, respectively. The functional impact of the mutations on the protein was predicted as described [26].

### Inference of transcription factor activity

Using the TCGA breast cancer dataset [21], E2F1 transcription factor activity was computationally inferred for each sample, as previously described [28, 29].

### Chromatin immunoprecipitation (ChIP) assays

ChIP assays were performed using the SimpleChip Enzymatic Chromatin IP kit (Cell Signaling Technology, 9003), as per manufacturer’s instructions. MCF7 breast cancer cells were cultured in 20cm tissue culture plates. At 85-90% confluence, 540μl of 37% formaldehyde was added and plates were shaken at ∼20rpm for 10min at room temperature (RT) to crosslink proteins to DNA. Two milliliters of 10x glycine were added, plates were shaken (∼20rpm, 5min, RT), media was removed and cells were washed twice with 10ml ice-cold PBS. Two milliliters of ice-cold PBS-PIC (1:500 PIC in PBS) were added and cells were scraped into the cold buffer. Cells were centrifuged (1500rpm, 5min, 4°C) and supernatant removed. Cells were resuspended in 1ml ice-cold Buffer A (3ml dH_2_O, 1ml 4xBuffer A, 2μl 1M DTT, 8μl PIC), incubated on ice for 10min and inverted every 3min to mix. Cells were centrifuged (3000rpm, 5min, 4°C) and supernatant was removed. Cells were resuspended in 1ml ice-cold Buffer B (3.3ml dH_2_O, 1.1ml 4xBuffer B, 2.2μl 1M DTT), centrifuged (3000rpm, 5min, 4°C) and supernatant was removed. Cells were resuspended in 100μl ice-cold Buffer B, 0.5μl of micrococcal nuclease was added and mixed by inverting several times. Cells were incubated at 37°C for 20min and inverted every 5min to mix. Next, 10μl of 0.5M EDTA was added and mixed by inverting. Digestion was stopped on ice for a few seconds. Cells were centrifuged (13,000rpm, 1min, 4°C), supernatant was removed, cells were resuspended in 500μl 1x ChIP buffer containing PIC and PMSF (100μl 10x ChIP buffer, 900μl dH_2_O, 2μl PIC, 10μl PMSF 100mM) and incubated on ice for 10min. Samples were sonicated for 6min (30sec pulse, 30sec wait on ice), centrifuged (10,000rpm, 10min, 4°C) and supernatant was stored at -80°C. To each 450μl sample, 20μl of protein G magnetic beads was added and rotated for 1hr at 4°C. Beads were removed following a brief spin. The respective antibodies were added and incubated overnight at 4°C with rotation. In addition to normal rabbit IgG (negative control, kit component), the antibodies used were against E2F-1 (C-20) X (2μg per sample, sc-193 X, Santa Cruz, VWR) and Histone H3 (positive control, kit component). To each sample, 30μl of protein G beads was added and incubated for 2hr at 4°C with rotation. Samples were placed in a magnetic separation rack for 2min and supernatant was removed. Samples were washed 3 times: each with 1ml of low salt wash buffer (1ml 10x ChIP buffer, 9ml dH_2_O), 5min incubation at 4°C with rotation and incubation in the magnetic separation rack for 2min before discarding the supernatant. Samples were washed with 1ml of high salt wash buffer (400μl 10x ChIP buffer, 3.6ml dH_2_O, 280μl 5M NaCl), incubated at 4°C for 5min with rotation and incubated for 2min in a magnetic separation rack before discarding the supernatant. Chromatin was eluted from the beads by incubation in 150μl of 1xChIP elution buffer (500μl 2xChIP elution buffer, 500μl dH_2_O) at 65°C for 30min with shaking at 13,000rpm in a thermomixer. Supernatant was transferred to a clean tube and cross-links reversed by adding 6μl 5M NaCl and 2μl Proteinase K and 2-hour incubation at 65°C. Following addition of 5 volumes of DNA binding buffer per 1 volume of sample, samples were centrifuged onto spin columns (14000rpm, 30sec). Flow-through was discarded, 750μl of DNA wash buffer was added to the spin columns, which were then centrifuged (14000rpm, 30sec) and flow-through was discarded. After an additional centrifugation step, 50μl of DNA elution buffer was added to the column, the column was centrifuged (14,000rpm, 30sec) and the eluted DNA was stored -20°C. The DNA was used for PCR and the products were run on a 2% agarose gel. The primers used were: CENPI F: 5’-gga acg cca gcc aat cag ctg ac-3’, R: 5’-ccc gcc acc tct agc caa tca gg-3’; RRP8 (negative control): F: 5’-CTT GGG ACT CAG GAG AAG TG-3’, R: 5’-AAC CAA AGC GTG ACA GCC AG-3’.

### Chromosome counting

The ER+ breast cancer cell line MCF7 was transfected with the plv411g expression vector (which contains an internal ribosomal entry site (IRES), followed by the GFP cDNA), either empty or containing the CENPI cDNA, using lipofectamine 3000 reagent (Thermo-Fisher). Four days after transfection, cells were fixed with 4% paraformaldehyde and permeabilized in 0.5% Tween-20 in PBS (PBS-T). Cells were blocked in 1% BSA, 300mM glycine in PBS-T and exposed to a primary antibody to the centromere protein CENP-A (1:100, Abcam, ab13939) diluted in 1% BSA in PBS-T buffer for 1 hour at room temperature (RT). Secondary antibody (1:400, AlexaFluor594) was diluted in 1% BSA in PBS and incubated for 1 hour at RT in the dark. Cells were counterstained with DAPI and mounted onto Single Frost 76×25mm slides (Labtek, 7105). GFP-positive cells were imaged in z-stacks using Spinning Disk Confocal Microscopy. Z-stack images were merged and the chromosome numbers per cell were obtained by counting the number of CENP-A signals using the cell counter function in ImageJ/Fiji software.

### CIN70 analyses

Using the RSEM-normalized data from the TCGA breast cancer RNAseq V2 dataset [21], the CIN70 score for each patient sample was calculated by averaging the normalized expression level of the 70 genes included in this signature [33]. The Pearson R^2^s for linear regression were also computed for the CIN70 score and each of the individual CIN70 genes, as well as for CENPI. The strength of CENPI as a marker for chromosome instability was benchmarked against the strength of the CIN70 genes by comparing and ranking the respective R^2^s.

### Somatic copy number variation analyses

TCGA breast invasive carcinoma processed (Level 3) Affymetrix Genome Wide SNP6.0 Array data and the associated clinical data were downloaded from the TCGA data portal [21]. Copy number data were post-processed by GISTIC2.0 using a threshold of >0.2 for amplification and ≤0.2 for deletion. Somatic copy number variations (CNVs) were determined by subtracting germline CNV from tumor CNV data aligned to hg19 using Python3 (www.python.org). Segments overlapping with the centromeres (according to UCSC genome browser for hg19 human reference build) were discarded from the CNV file. For each sample in the CNV file and for each chromosome arm, total segment lengths – regardless of segment mean – were summed. For each chromosome arm, the length of amplification or deletion (with |segment mean|>0.2) was summed. Per chromosome arm, fractions amplified or deleted were calculated by dividing the sum of the segment lengths amplified or deleted by the total length of segments. Whole-chromosome CNV calls were determined by summing arm-level data for each chromosome. For each tumor sample, individual chromosome arms or whole chromosomes were scored as gained or lost, if at least 90% of the arm or whole-chromosome was gained or lost (absolute log2 copy-number threshold ≥0.2), respectively. Tumors were considered aneuploid if at least one whole chromosome was gained or lost. Using the clinical data files, only ER+ tumors were assessed. Gene expression levels, i.e., mRNA levels, in the respective samples were retrieved from the TCGA breast carcinoma RNAseq V2 dataset as described above.
